# Chromatin Interaction and Histone Mark Signatures Associated With *TBXT* Expression in Metastatic Lung Cancer

**DOI:** 10.1002/gcc.70041

**Published:** 2025-03-18

**Authors:** Reuben M. Yaa, Brian M. Schilder, Rafael D. Acemel, Fiona C. Wardle

**Affiliations:** ^1^ Randall Centre for Cell & Molecular Biophysics King's College London London UK; ^2^ UK Dementia Research Institute Imperial College London London UK; ^3^ Department of Brain Sciences Imperial College London London UK; ^4^ Centro Andaluz de Biología del Desarrollo (CABD) Consejo Superior de Investigaciones Científicas/Universidad Pablo de Olavide Seville Spain

**Keywords:** 4C‐seq, A549, cells, ChIP‐seq, epigenomics, H358, H460, lung cancer, *TBXT*

## Abstract

**Background:**

*TBXT,* a member of the T‐box transcription factor family, drives epithelial‐to‐mesenchymal transition in the metastasis of some cancers. However, the relationship between the epigenetic regulatory landscape and its expression in lung cancers remains elusive.

**Methods:**

Circularized chromosome capture combined with sequencing (4C‐seq) was employed to analyze physical chromatin interactions at the *TBXT* loci in the lung cancer cell line H460, a high *TBXT‐expressing* cell line, compared to H358 and A549, which do not express *TBXT*. To define the regulatory landscape, the targeted *TBXT* chromatin interactions were integrated with histone modification profiles from respective cells, followed with motif analysis.

**Results:**

Our analysis identified distinct patterns of potential *cis*‐regulatory elements (pCREs) associated with the *TBXT* promoter, with increased near‐*cis* pCRE enrichment in the *TBXT‐expressing* cells. Integration of pCREs with epigenetic histone modification revealed two unique pCREs in *TBXT‐expressing* H460 cells enriched with the active histone mark H3K27ac, harboring binding sites for transcription factors of the forkhead box, zinc finger, and musculoaponeurotic fibrosarcoma families that are linked to cancer metastasis.

**Conclusion:**

Our findings shed light on active chromatin interactions with *TBXT* expression in lung cancers, pointing to specific DNA elements and regulatory proteins that may be involved. This knowledge paves the way for understanding *TBXT* expression dynamics at the onset and progression of metastatic cancers.

## 1 Introduction

Lung cancer is the deadliest cancer in the world, with approximately 1.8 million deaths in 2020 [1]. If diagnosed and treated at early stages, 5‐year survival rates for lung cancer patients can be greater than 50%; however, if diagnosed at late stages, when the cancer has metastasized to other organs, 5‐year survival rates can be lower than 4% [2], 2016). Understanding the factors that trigger metastasis and how these events are controlled is important for understanding how to prevent and treat late‐stage lung cancer.

One factor of interest is the T‐box transcription factor, TBXT (also known as T or Brachyury). Although studied for decades in the context of embryonic development [3, 4], more recent evidence indicates TBXT also plays an active and central role in lung cancer progression and metastasis [5].

Studies have shown that a subset of non‐small cell lung carcinomas (NSCLC) express high levels of *TBXT* at the mRNA and protein levels, while normal adult tissues (with the exception of the testis and thyroid) do not express *TBXT* [6, 7, 8, 9]. High levels of TBXT in these tumors are correlated with late disease stages, distant metastases, and reduced survival [7, 10, 11, 12]. This finding is consistent with in vitro assays showing that high levels of TBXT cause upregulation of markers of epithelial‐to‐mesenchymal transition (EMT) and increase motility and invasiveness of cancer cells, while knocking down *TBXT* limits tumor metastasis in in vivo models [7, 13], suggesting that TBXT drives an invasive phenotype. Other studies have shown that high levels of TBXT mediate resistance to chemo‐, radio‐, and immunotherapy [11, 13, 14]. Taken together, these findings, and similar evidence indicating that high levels of TBXT mediate cancer progression in other tumor types including breast and prostate cancer, have made TBXT an attractive therapeutic target [15]. However, despite this role in cancer progression, our understanding of how *TBXT* expression is activated and maintained in lung cancer cells is incomplete, which limits advances in targeted therapies.

Although fibroblast growth factor receptor 1 (FGFR1) has been implicated in activating *TBXT* expression in lung cancer through MAPK signaling [16], the genomic *cis*‐regulatory regions that mediate this activation are unknown. On the other hand, *TBXT* expression in the rare cancer, chordoma, is regulated via a super‐enhancer (SE) downstream of the *TBXT* gene, which binds TBXT protein to maintain its own expression [17, 18]. However, whether such epigenetic regulation characterizes *TBXT* expression in lung cancer is unknown.

To begin to address this gap in knowledge about *TBXT* expression regulation, we have used circularized chromosome conformation capture coupled with next‐generation sequencing (4C‐seq) [19, 20] to capture, at high resolution, genomic regions that interact with the *TBXT* promoter, which may represent potential *cis*‐regulatory elements (pCREs) in lung cancer cell lines. For this, we compared interactions in a *TBXT‐expressing* lung cancer cell line, H460, which is derived from pleural fluid of a patient with large cell cancer of the lung [21] with two non‐small cell lung cancer (NSCLC) adenocarcinoma cell lines, A549 and H358, which have non‐metastatic properties and do not express *TBXT* [8, 16]. Previous evidence has shown higher resistance to EGF receptor (EGFR) inhibition, immune‐mediated lysis, as well as conventional chemotherapies and radiation in H460 cells compared to A549 and H358 cells [8, 16]. H460 lung cancer cells also express higher levels of MYC compared to A549 [22] and exhibit early and high invasiveness [23]. This suggests H460 is more tumorigenic and malignant than the other two. We identified common and unique interactions of genomic regions with the *TBXT* promoter in *TBXT* vs. non‐*TBXT‐expressing* environments, which we grouped into shared, gained, or lost in relation to H460 cells and assessed their enrichment in various histone marks. Typically, histone marks such as H3K27me3 and H3K9me3 are modifications associated with transcriptional silencing [24, 25], while H3K27ac and H3K4me3 are associated with active gene expression chromatin state and, in addition, have been shown to be elevated in cancer [25, 26] and at various cancer risk genes [17]. A proportion of gained interactions that were exclusively found in the *TBXT‐expressing* environment were enriched with the active H3K27ac epigenetic mark compared to lost interactions in H460 cells. These interacting regions were enriched with binding motifs for forkhead box (FOXH1, FOXQ, FOXJ3, FOXO4), zinc finger (ZNF214, ZNF134, ZNF549 and ZNF563) and musculoaponeurotic fibrosarcoma proteins (MAFK, MAFB, MAFG, MAFF and MAF), which have all been implicated in cancer metastasis [27, 28, 29, 30, 31]. Our analysis points to a regulatory program associated with *TBXT* expression in metastatic lung cancer.

## 2 Materials and Methods

### 2.1 Cell Culture

Human lung cancer cell lines were obtained from ATCC (H460) or were a kind gift of Prof. Maddy Parsons, King's College London (H358 and A549). All the lines were authenticated by short tandem repeat analysis (Eurofins) and regularly checked for *Mycoplasma* contamination by PCR. H460 and H358 cells were maintained in RPMI 1640 (Sigma‐Aldrich; R8758) and A549 cells were maintained in DMEM (Sigma‐Aldrich; D5796), all supplemented with 10% (v/v) fetal bovine serum (FBS; Sigma‐Aldrich; S0615) and 100 U/mL penicillin/0.1 mg/mL streptomycin (Sigma‐Aldrich; P4333) and maintained in a humidified 37°C incubator with 5% CO_2_. For immunofluorescence, cells were seeded on glass coverslips in 24‐well plates.

### 2.2 Reverse Transcriptase PCR

Total RNA was extracted using the Direct‐Zol RNA miniprep kit (Zymo Research; R2052) from 0.5 × 10^6^ cells resuspended in cold Trizol (ThermoFisher Scientific; 15 596 026). cDNA was synthesized using the High‐Capacity cDNA Reverse Transcription Kit (ThermoFisher Scientific; 4 368 814). *TBXT* and *GAPDH* were detected using GoTaq DNA polymerase (Promega) with the following primers: *TBXT*_E7‐F (5′‐GGGTGGCTTCTTCCTGGAAC), *TBXT*_E7‐R (5′‐TTGGAGAATTGTTCCGATGAG), *GAPDH*‐F (5′‐AGATCCCTCCAAAATCAAGTGG) and *GAPDH*‐R (5′‐GGCAGAGATGATGACCCTTTT). PCR products were amplified for 25 cycles of 30 s at 95°C, 30 s at 60°C, and 30 s at 72°C, followed by one cycle of 72°C for 5 min.

### 2.3 Western Blot

A total of 0.5 × 10^6^ cells were pelleted, lysed, and denatured by heating at 95°C for 10 min in 2X Laemmli sample buffer (Bio‐Rad; 1 610 737) containing 10% 2‐mercaptoethanol (Aldrich; M6250). The proteins were resolved on a 12% SDS polyacrylamide gels and blotted on PVDF membrane (ThermoFisher Scientific; 88 518) using the semi‐dry transfer method (Trans‐Blot TurboTM, BioRad; 1 704 150). The membranes were incubated for 1 h at room temperature in blocking buffer (1X phosphate buffered saline PBS; Thermo Fisher Scientific; BR0014G), 1% Tween 20 (VWR; 0777‐1 L), 5% nonfat milk (VWR; 22 012) and then incubated overnight at 4°C in a 1:500 dilution of anti‐TBXT (R&D Systems; AF2085, LOT NO: KQP0618021) or a 1:1500 dilution of anti‐histone H3 (Abcam; ab1791) in blocking buffer. The membranes were then washed 3 times for 5 min in PBST (1X PBS, 1% Tween 20) and incubated in a 1:5000 dilution of HRP‐conjugated anti‐rabbit IgG (Sigma‐Aldrich, AP182P; LOT NO: 3130473) or anti‐goat IgG (Sigma‐Aldrich, AP180P, LOT NO: 3157520) in blocking buffer at room temperature for 2 h. After washing three times for 5 min in PBST, the proteins were detected using SuperSignal West Pico PLUS Chemiluminescent substrate (ThermoFisher Scientific; 34 577) according to the manufacturer's protocol.

### 2.4 Immunofluorescence Microscopy

Cells on coverslips were fixed for 20 min in 4% paraformaldehyde (Sigma‐Aldrich, 158 127), then permeabilized and blocked in 10% bovine serum albumin (BSA; Sigma‐Aldrich, A9418) and 0.3% Triton X‐100 (Sigma‐Aldrich, ×100) in 1× PBS. The cells were then incubated overnight at 4°C in a 1:800 dilution of anti‐TBXT (R&D systems; AF2085, LOT No. KQP0618021) in dilution buffer (1× PBS, 1% BSA, 1% Rabbit Serum (Thermo Fisher Scientific, 16 120 099), 0.3% Triton X‐100). The cells were washed three times for 5 min in wash buffer (1× PBS, 0.1% BSA) then incubated at room temperature for 1 h with a 1:5000 dilution of anti‐goat IgG‐Alexa Fluor Plus 594 (ThermoFisher Scientific, A32758) in dilution buffer. After washing again, the cells were incubated for 10 min in a 1:1000 dilution of DAPI (Sigma‐Aldrich, MBD0015‐1ML) in 1× PBS, washed in 1× PBS then distilled water, and mounted on slides using Vectashield anti‐fade mounting medium (Vector Laboratories, H‐1000‐10). Images were taken using a Zeiss Axiovert 200 M fluorescence microscope with a 20×/0.85 ph 1 objective lens. Graph‐based optical intensities from the immunofluorescence (IF) images for TBXT and DAPI were obtained by ImageJ 1.49v. Intensities were expressed as mean ± SD of all the cells per cell line, and intensity differences between cell lines were assessed using the Wilcoxon rank‐sum test.

### 2.5 4C‐Seq Library Preparation and Sequencing

Two biological replicate libraries were prepared for each of the lung cancer cell lines according to a previous protocol [19, 20]. Briefly, a total of 10 million cells were crosslinked in 2% paraformaldehyde followed by quenching. The recovered pellet was lysed in cold lysis buffer (50 mM Tris–HCl pH 7.5, 150 mM NaCl, 5 mM EDTA, 0.5% Igepal CA‐630 Sigma‐Aldrich, I8896), 1% Triton X‐100, and 1× cOmplete protease inhibitors (Roche, 11 245 200). Chromatin was then digested overnight at 37°C using *Nla*III endonuclease (New England Biolabs, R0125L). Digestion was stopped by heat inactivation, and fragmented nuclei were ligated with T4 DNA ligase (NEB, M0202) at 16°C overnight. Circularized chromatin was de‐crosslinked using proteinase K (20 mg/mL) (NEB, P8107S) at 65°C overnight, then treated with RNase A (20 mg/mL) (Thermo Fisher Scientific, 12 091 021) for 45 min at 37°C. Chromatin was purified using phenol‐chloroform precipitation and digested at 37°C overnight with *Dpn*II (NEB, R0543S) followed by enzyme heat inactivation. The samples were ligated at 16°C using T4 DNA ligase (NEB, M0202) to form 4C chromatin circularized templates. These templates were purified by centrifugation using Merck Millipore Amicon ultra‐15 centrifugal filter units (Merck, 106 608 072). Using the chromatin circularized templates, *TBXT* circularized chromatin was captured by PCR using *TBXT* inverse primers overlapping the ligation junction as follows: PCR Reactions were prepared in eight x 25 μL volumes using Q5 polymerase (NEB, M0491L) and inverse sequencing primers (see Table S1) with the following cycling procedure: one cycle of 98°C for 30 s, then 30 cycles of 98°C for 10 s, 57°C for 30 s, and 72°C for 30 s. Indexed DNA amplicons products were pooled, purified using High Pure PCR product purification kit (Roche, 11 732 668 001) and 0.8:1 ratio with AMPure XP reagent (Agencourt, AMPure XP). The product concentrations were assessed using Qubit dsDNA BR assay Kit (Thermo Fisher Scientific, Q32850). The libraries were deep sequenced as 150 bp on a HiSeq X Ten at BGI, Hong Kong.

### 2.6 4C‐Seq Analysis

Reads were demultiplexed, trimmed to 30 bp, and aligned using Bowtie v1.2.2 aligner [32] to a *Nla*III‐*Dpn*II fragment map of the GRCh38 (hg38) *Nla*III‐*Dpn*II using *‐m* 1 and default settings. Reads falling in fragments flanked by the same restriction enzyme site, in fragments smaller than 40 bp, or in fragments within ±5 kb from the viewpoint were removed. The aligned reads were then transformed to reads per *Nla*III fragment ends and smoothened using a 30‐fragment mean running window. Total read counts in the fragments were quantified in 5 kb bins 1 Mb either side of the viewpoint. The counts were used to assess the reproducibility of biological replicates and sample clustering using correlation analysis. 4C profile information for the replicates was subsequently used to identify fragments interacting with the viewpoint using monotonic regression in the peakC peak caller [33] and assessment of the fragment local coverage score [34]. Fragments were considered to interact with the viewpoint if they satisfied all of the following criteria: (1) appeared in both replicates, (2) had a monotonic regression value above 2.5% of the value of the monotonic regression at the viewpoint, (3) had a monotonic regression value high enough to ensure an adequate signal‐to‐noise ratio, and (4) had a local coverage score in the upper quartile. This was fitted for every fragment 1 Mb either side of the viewpoint. *TBXT* promoter‐interacting fragments that were not more than 40 bp from each other were merged using bedtools v2.27 [35] to form interacting regions (pCREs) and plotted as an arachnogram to generate an intuitive *cis*‐interaction visualization landscape.

### 2.7 Linear Analysis of Differential Chromatin Interactions

The pCREs were processed for the assessment of changing chromatin interactions to the *TBXT* locus between the conditions as applied previously [24]. Since lung normal tissues do not express *TBXT* [6], interactions in the non‐*TBXT‐expressing* cells (A549 and H358) were used as a baseline control reference. Using this approach, interactions exclusively found in H460 and not in A549 or H358 cells were classified as gained in H460 cells, whereas those only present in A549 and/or H358 were classified as lost from H460 cells. Interactions present in both conditions were classified as shared. The pCREs were subsequently annotated using HOMER v4.11 [36]. The distribution of shared, gained, and lost pCREs across the genome was visualized as smoothed densities, based on their absolute distance from the viewpoint.

### 2.8 4C Integration With Epigenetic Data

#### 2.8.1 ChIP‐Seq Analysis

The pCREs were integrated with publicly available ChIP‐seq datasets for different histone modifications in A549, H358, and H460 cells. These datasets were reprocessed from the raw files using a standardized pipeline to ensure comparability. Raw fastq files of H3K27me3, H3K9me3, H3K27ac, and H3K4me3 were retrieved from Sequence Archive Reads (SRA; Table S2). Single‐end reads were aligned using Bowtie2 [32] followed by post‐alignment indexing and filtering using SAMtools [37]. The mapped files were subsequently processed using HOMER v4.11 [36] and tracks visualized in the UCSC browser. Peaks were called using MACS3 [38] implemented via the R package MACSr [39] within PeakyFinders (https://github.com/neurogenomics/PeakyFinders). The default cut‐off parameter was used (cut‐off = 5), corresponding to only using bedGraph reads with *p*‐values ≤ 1 × 10^−5^.

#### 2.8.2 Epigenetic Enrichment Analyzes

The *TBXT* pCREs were converted into a named list of GenomicRanges objects [40]. Permuted overlap enrichment tests (10 000 permutations per comparison) were conducted between all pairwise combinations of gained and lost pCRE types and reference ChIP‐seq peaks. This was performed for 2 pCRE types (gained or lost) compared against 4 histone marks across 3 cell lines with up to 2 histone replicates each, yielding 36 tests total. Enrichment tests were conducted using an extension of the regioneR package [41] implemented within echoannot [42]. The false discovery rate (FDR) was calculated according to the Benjamini‐Hochberg procedure to correct for multiple testing [43], and results at FDR < 0.05 were considered non‐significant. As H3K27me3 signals at the *TBXT* promoter in H358 cells were very low (Figure S2A) and there was a discrepancy in the clustering of H358 ChIP signals with the rest of the ChIP data (Figure S2B) epigenetic enrichment with *TBXT* interacting regions in the H358 cell data was omitted. However, the correlation of the whole dataset was performed (Figure S3A).

#### 2.8.3 Epigenetic Correlation Analyzes

All the *TBXT* interacting regions and ChIP‐seq peaks falling within a +/−1 Mb window surrounding the *TBXT* promoter viewpoint were re‐binned into 1 708 060 bins of 100 bp across chromosome 6 using EpiCompare [44]. Bins were filled with standardized percentiles derived from loop/peak strength. Bins with a row sum of 0 (i.e., no sample overlapped with that bin) were then removed, leaving a 3307‐bin x 17‐sample sparse matrix (containing 89.3% 0 s). Pearson's correlation (*r*) was then computed between each pair of samples. The resulting correlation matrix was subsequently plotted as a heatmap (Figure S3B; for an interactive version of the heatmap, please see: https://bschilder.github.io/TBXT_loops/code/heatmap).

#### 2.8.4 Motif Analysis

De novo motif discovery of two gained pCREs enriched with H3K27ac in H460 cells was performed using a component of Regulatory Sequence Analysis Tools (RSAT) v1.169 [45]. Motifs were predicted using oligomer lengths of 6 and 7. Enriched motif sequences were clustered to a reduced redundancy position‐specific scoring matrix that was used for searching curated transcription factor binding motifs in the HOmo sapiens COmprehensive MOdel COllection (HOCOMOCO Human v11 CORE) motif database (retrieved in 2024) [46] using the TomTom motif comparison tool in the MEME suite 5.3.3 [47].

## 3 Results

### 3.1 Chromatin Interaction Differences Between *TBXT* Expression Conditions

**FIGURE 1:**
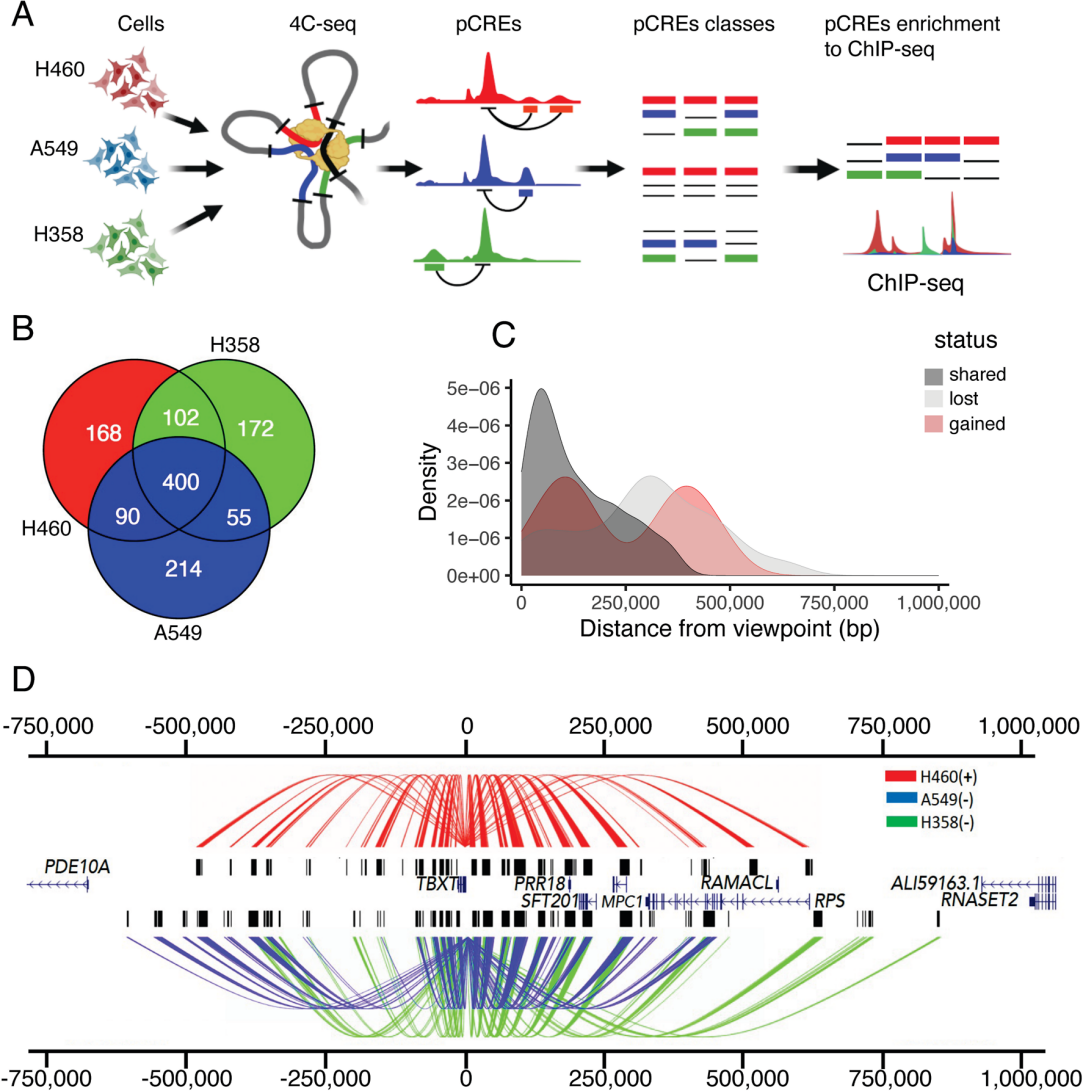
The *TBXT* promoter genome‐wide contacts in non‐ and *TBXT‐expressing* cells. (A) 4C‐seq library preparation from H358, A549, and H460 cells. The 4C‐seq data was analyzed, and 4C interacting regions identified as pCREs were classified as either shared, gained, or lost, and their enrichment with histone ChIP‐seq data was assessed. (B) Overlap of shared and unique pCREs between the cell lines. (C) Density plots of shared, gained, and lost pCREs in relation to the *TBXT* expressing line (H460). (D) Arachnograms highlighting significant interactions (pCREs) with the *TBXT* promoter. Lines originate from the viewpoint (*TBXT* promoter) and indicate regions interacting with the viewpoint (pCREs), shown as black bars. (+) indicates cell lines with high levels of *TBXT* expression, (−) indicates cell lines with no expression of *TBXT*. The gene symbols are shown next to the genes.

We conducted high‐resolution 4C‐seq (Figure 1A) to detect chromatin interactions with the *TBXT* promoter (viewpoint) in lung cancer cells that expressed high levels of *TBXT* mRNA and protein (H460 cells) and those that did not express detectable levels of *TBXT* mRNA or protein (A459 and H358 cells) (Figure S1). We reasoned this approach might allow us to detect variations in chromatin interactions with the *TBXT* promoter that are associated with these *TBXT* expression disparities. The 4C‐seq profiles (Figure S2A) had reproducible consistency between cell line replicates, with high pairwise correlation between the cells. H358 and A549 cells, the *TBXT* non‐expressers, were slightly more similar to each other than to H460 cells (Figure S2B,C).

On quantifying the interactions, our analysis revealed 760, 759, and 729 genomic regions that interact with the *TBXT* promoter (referred to here as putative CREs; pCREs) in H460, H358, and A549 cells respectively, within 1 Mb either side of the *TBXT* promoter. Of these, one‐third (400 out of a total of 1201 pCREs) are shared between all cells, while an additional 192 are shared between H460 cells and one other cell line (giving a total of 592 pCREs referred to here as “shared” between H460 cells and at least one other in this study; Figure 1B), indicating there is considerable similarity in genomic interactions with the *TBXT* promoter in the different lung cancer cell lines. Notwithstanding this similarity, we also observed cell line‐specific *TBXT* interactions that were present only in H460 cells (168 pCREs, referred to as “gained” in H460 cells) or only present in one or both of the two *TBXT‐negative* cell lines, A549 and H358 (441 pCREs, referred to as “lost” relative to H460 cells; Figure 1B). All 168 gained pCREs are in non‐coding regions, with only two that are in the promoter region immediately upstream (within −600 bp) of the Transcription Start Site (TSS) of *RPS6KA2*, while the majority of lost pCREs (436/441) are also in non‐coding regions, with one also within −1 kb of the *RPS6KA2* TSS (Table S3).

Mapping the distance and distributions of each class revealed that shared pCREs are concentrated in near‐*cis* within 0.2 Mb from the *TBXT* promoter, and gained pCREs are similarly observed in near‐*cis* as well as medium‐cis (between 0.2 and 0.5 Mb), while pCREs present only in TBXT negative cell lines are observed in medium‐ and far‐*cis* (beyond 0.5 Mb; Figure 1C). Consistent with this, linear genome representation of the *TBXT* promoter‐pCRE interactions by cell line type revealed a broader span from the *TBXT* promoter viewpoint in H358 and A549 cells compared to H460 cells, where pCREs are primarily concentrated within 0.5 Mb of the viewpoint (Figure 1D).

### 3.2 Associating Chromatin Interactions With Epigenetic Marks

**FIGURE 2:**
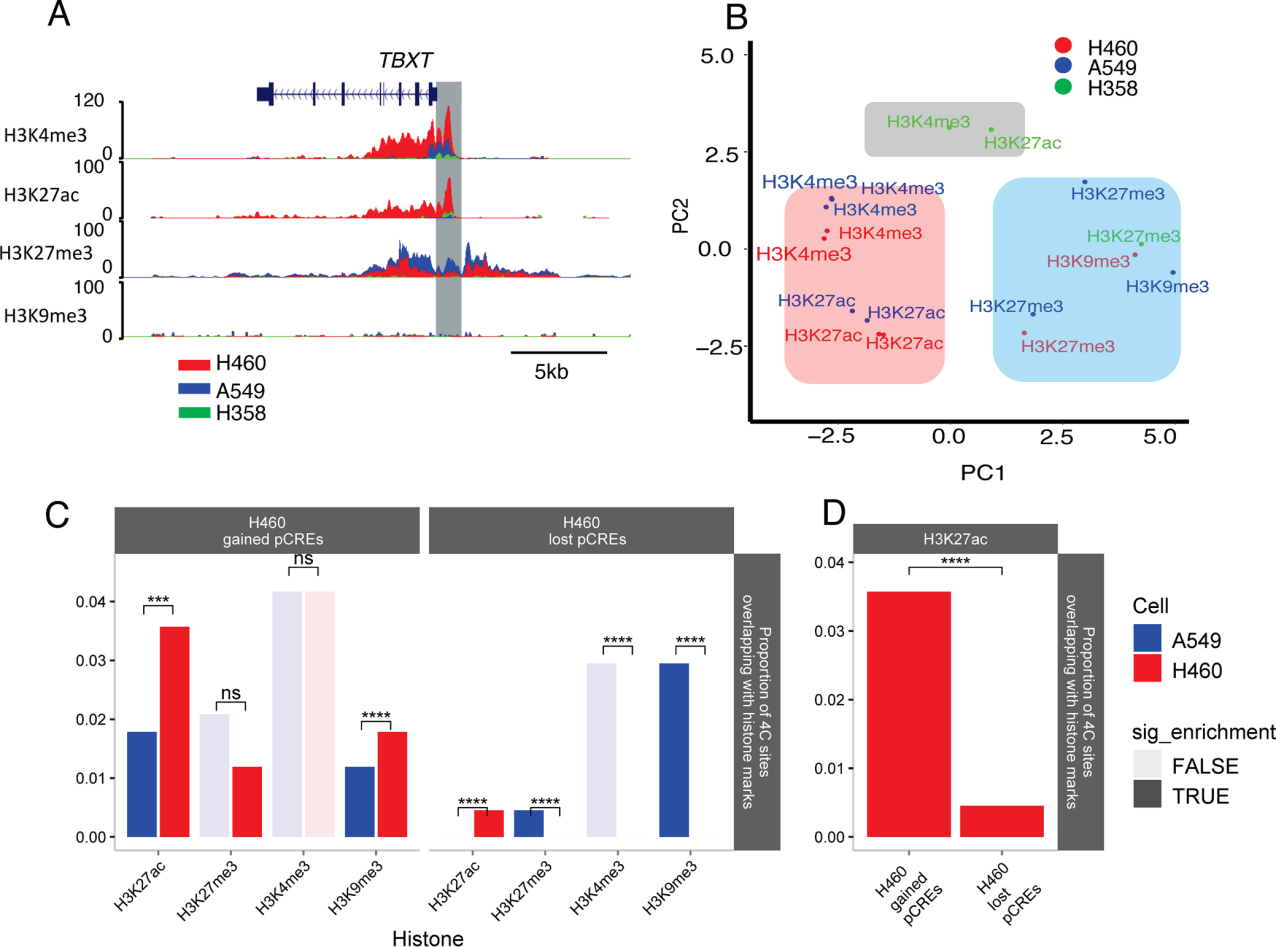
Overlap and enrichment of *TBXT* pCREs with histone marks at the *TBXT* locus. (A) Overlay of merged ChIP‐seq histone modifications at the *TBXT* locus (immediate upstream region highlighted in gray) for the H460 (red), A549 (blue) and H358 (green) lung cancer cells. (B) PCA clustering of the ChIP‐seq peaks at the *TBXT* locus. The active marks H3K27ac and H3K4me3 are highlighted in faint red, while the repressive marks H3K27me3 and H3K9me3 are highlighted in faint blue. Active marks from H358 cells highlighted in gray form a cluster separate from the rest of the active marks. (C, D) Proportional overlap of pCREs with the indicated histone marks by permuted overlap enrichment tests (10 000 permutations per test) between ChIP‐seq peaks (4 histone marks × 2 cell‐lines) and each *TBXT* pCRE type with multiple repressive (H3K27me3, H3K9me3) and active regulatory marks (H3K4me3, H3K27ac) In experiments where multiple replicates existed, mean FDR and mean *z*‐scores were computed after conducting proportional enrichment tests with each replicate. Where the proportional overlap is not statistically significant (FALSE), the corresponding bar in the graph is displayed in faint color. Conversely, significant overlap (TRUE) is represented by bold‐colored bars. Asterisks indicate significant difference between cell lines (****: FDR < 0.0001, ***: FDR < 0.001). (C) Enrichment of histone marks at pCREs gained in the H460 cell line (gained pCREs) and lost in the H460 cell line (lost pCREs). (D) H3K27ac enrichment difference between H460 gained and lost pCREs.

We wanted to know if changes in pCREs between cell lines were associated with changes in histones at the genomic locus, so we assessed the proportional overlap of pCREs between *TBXT‐expressing* conditions and H3K27ac, H3K4me3, H3K27me3, and H3K9me3 marks using publicly available ChIP‐seq data for A549, H358, and H460 cells (Table S2). While we could not directly verify *TBXT* expression in the cells used in these ChIP‐seq datasets, we investigated the occupancy of H3K27ac and H3K4me3 (active gene expression) and of H3K27me3 and H3K9me3 (inactive transcription) at the *TBXT* locus in the datasets. In the H460 ChIP‐seq datasets, the *TBXT* locus was distributed with H3K27ac and H3K4me3 marks similar to the patterns observed in other *TBXT‐expressing* lines and tissues [17, 18, 48], suggesting active transcription of *TBXT* in the H460 cells used to generate the ChIP‐seq data. Conversely, in the A549 and H358 datasets, H3K27ac and H3K4me3 marks are absent or reduced, while A549 cells show an increase in H3K27me3 at the *TBXT* transcription start site, suggesting a lack of *TBXT* expression in these A549 and H358 cells (Figure 2A). However, the H3K27me3 signal in H358 cells at the *TBXT* promoter was too low to be used in this or further analysis. Hence, the *TBXT* expression profiles of the cells used to generate the ChIP‐seq datasets were comparable to the *TBXT* expression profiles of cells from which we generated 4C‐seq data (Figure S1). Interestingly, analysis of histone ChIP‐seq data 1 Mb either side of *TBXT* clustered primarily by histone mark followed by cell line (Figures 2B and S3A,B), indicating potential cell line variations in the regulation, particularly for genes around the *TBXT* locus. Interestingly, in contrast to chordoma cells, we also note that there is no evidence of a super‐enhancer in the *TBXT‐expressing* H460 cells. Having verified the datasets, we proceeded to evaluate the differential epigenetic enrichment at pCREs in the different *TBXT‐expressing* environments.

This analysis revealed an enrichment of the active histone mark H3K27ac at H460 gained pCREs in *TBXT‐expressing* H460 cells compared to non‐expressing A549 cells. On the other hand, there was a similar overlap of gained pCREs with H3K4me3 in both cell lines, although this overlap did not reach a statistical significance, and there was no enrichment of H3K4me3 between the cell lines (Figure 2C). Further, in line with their correlation with *TBXT* expression, the H460 gained pCREs exhibited lower enrichment of repressive histone marks (H3K27me3 and H3K9me3) compared to the active mark H3K27ac in H460 cells (Figure 2C). Within the repressive marks, H3K9me3 enrichment was unexpectedly higher in H460 cells compared to A549 cells (Figure 2C) possibly due to cell line‐specific variations in H3K9me3 function and/or heterogeneity within the H460 gained pCREs population. The other repressive mark, H3K27me3, exhibited a non‐significant reduction in H460 cells compared to A549 cells. Furthermore, statistical comparisons reveal a significantly higher association of H3K27ac with gained pCREs than lost pCREs in H460 cells (Figure 2D). Overall, the strong enrichment of H3K27ac at gained pCREs, i.e., regions that interact with the *TBXT* promoter in H460 cells but not non‐*TBXT‐expressing* cells, may suggest a role of these in *TBXT* expression regulation in H460 cells.

On the other hand, the epigenetic landscape of H460 lost pCREs displayed variable enrichment patterns compared to the gained pCREs. The repressive histone marks H3K27me3 and H3K9me3 displayed enrichment in these pCREs in A549 cells compared to H460 cells (Figure 2C). There was also enrichment of these pCREs with H3K27ac in H460 compared to A549. However, the overall enrichment levels for H3K27ac were lower than the enrichment observed for the repressive marks within these pCREs. These patterns could indicate a role for these lost pCREs, which interact with the *TBXT* promoter in A459 cells, in repression of *TBXT* expression in A549 cells.

**FIGURE 3:**
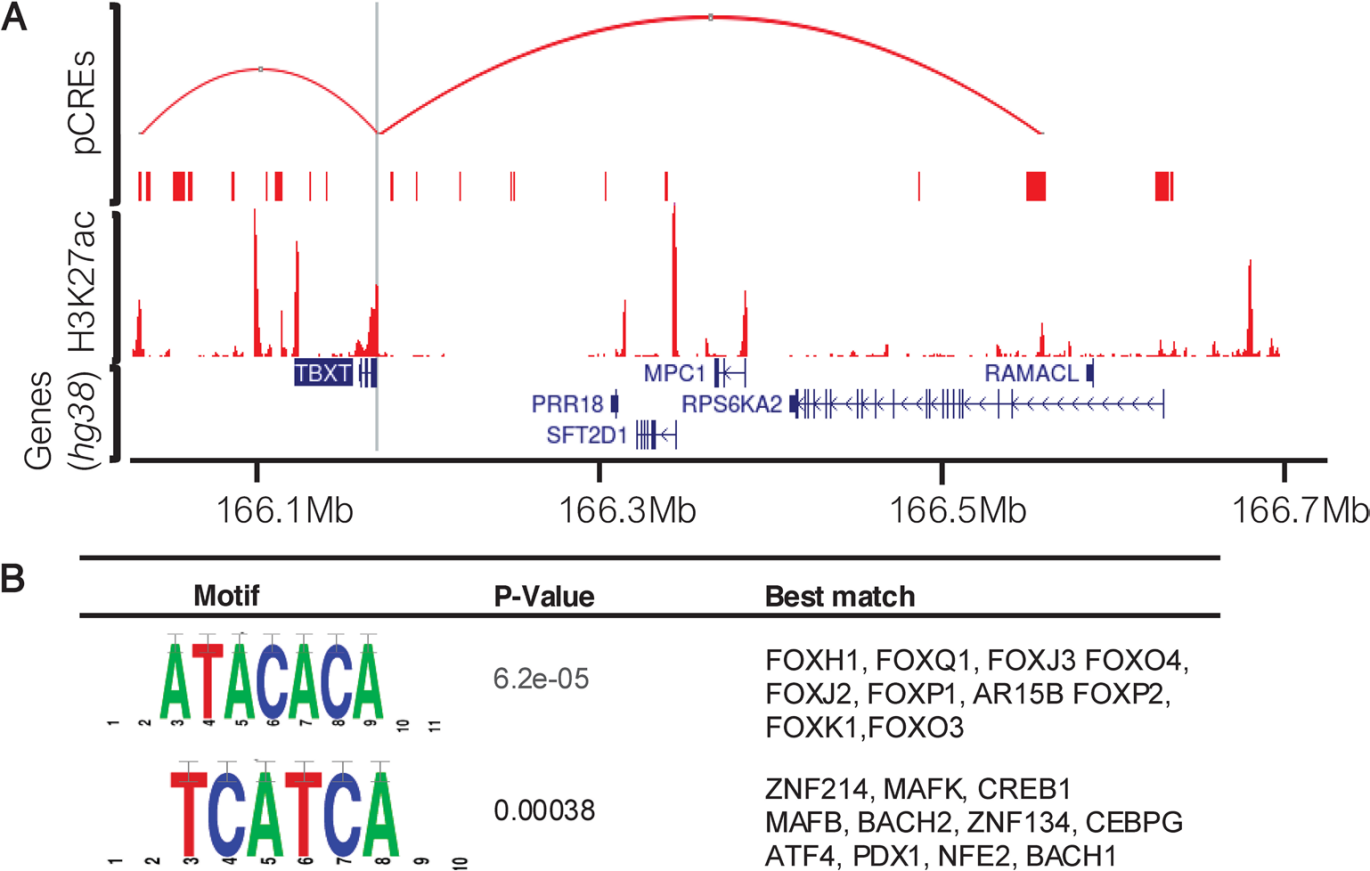
Multi‐track plot of H460‐gained loops that overlap with H3K27ac active histone marks. (A) Multi‐track plot showing the overlay of putative *TBXT* cis‐regulatory elements (pCREs) and H3K27ac active mark in H460 cells. The viewpoint is shown as a vertical gray line. The top track (pCREs) shows the H460 gained pCREs that were experimentally quantified in the present study on top of all pCREs from H460 (red bars). The second track (H3K27ac) shows merged normalized read counts of H3K27ac ChIPseq data for H460 (*n* = 2). The third track shows *TBXT* and other genes in the region. (B) Motifs (*p* < 0.05) detected de novo by RSAT and the top 10 TF best hits from (HOCOMOCO Human (v11 CORE)) motif database.

Given the enrichment analysis revealed H460 gained pCREs with stronger enrichment of the active histone mark H3K27ac in H460 cells compared to A549 cells and H460 lost pCREs (Figure 2C,D), we prioritized these pCREs to identify potential regulatory elements within them that drive *TBXT* expression. The selected pCREs (hg38, chr6:166029223–166030302 & chr6:166547780–166558507) are visualized in (Figure 3A), which also shows ChIP‐seq read densities for H3K27ac in *TBXT‐expressing* H460 cells. We conducted motif discovery in these selected pCREs to uncover potential transcription factors that might bind and regulate *TBXT* expression. Two motifs were identified (Figure 3B). Search on the reference motif database revealed enrichment of binding motifs for the transcription factors in the forkhead box family (FOXH1 *p* = 5.14 × 10^−4^, FOXQ1 *p* = 2.381 × 10^−3^, FOXJ3 *p* = 3.04 × 10^−3^, FOXO4 *p* = 4.05 × 10^−3^), zinc finger protein (ZNF214 *p* = 8.4 × 10^−4^, ZNF134 *p* = 9.37 × 10^−3^, ZNF549 *p* = 1.9 × 10^−2^ and ZNF563 *p* = 2.27 × 10^−2^) and musculoaponeurotic fibrosarcoma proteins (MAFK *p* = 8.20 × 10^−3^, MAFB *p* = 8.590 × 10^−3^, MAFG *p* = 1.29 × 10^−2^, MAFF *p* = 1.488 × 10^−2^ and MAF *p* = 1.68 × 10^−2^) (Figure 3B, Table S4). These findings point to the possibility that selected transcription factors in the forkhead box, zinc finger, and Maf family could potentially act as transcriptional regulators of *TBXT* expression through interaction with the *TBXT* enhancers that were identified in the current study.

## 4 Discussion

Previous research indicates the involvement of FGFR1 in activating *TBXT* expression in lung cancer via MAPK signaling [16], while a super‐enhancer downstream of *TBXT* is evident in chordoma [17, 18]. In this study, we present a *TBXT* epigenetic regulatory landscape from *TBXT‐expressing* and non‐expressing lung cancer cell lines. Our data indicate a substantial overlap of *TBXT* promoter –genome interactions among cell lines, implying a relatively stable chromatin conformation at the *TBXT* locus regardless of *TBXT* expression levels and cell lines. This is understandable as all the cell lines used herein were derived from lung tumors. The shared stable subset of chromatin interactions with the *TBXT* promoter in all cell lines is unlikely to influence the different expression of *TBXT* between the cell lines. However, we still found differential chromatin interactions to the *TBXT* locus between the cell lines, prompting an evaluation of their enrichment with active gene expression (H3K27ac and H3K4me3) and inactive transcription (H3K27me3 and H3K9me3) histone marks, which may infer their roles in *TBXT* expression.

Examining the proportional overlap enrichment between pCREs and histone marks provided insight into the features of different pCREs. Our analysis revealed a greater enrichment of H460 gained pCREs with the active gene expression mark (H3K27ac) and with diminished enrichment of the repressive mark H3K27me3 in the *TBXT* expressing environment. Thus, within *the TBXT* expressing environment, the *TBXT* promoter interacts with specific CREs enriched with active regulatory chromatin marks, which might suggest they act as enhancers to upregulate and/or maintain *TBXT* expression in H460 cells. Additionally, higher enrichment of repressive marks (H3K27me3) with H460 lost pCREs in non‐*TBXT* expressing lung cancer cells (A549) compared to H460 may indicate a role in the suppression of *TBXT* expression in the *TBXT* non‐expressing environment. However, the pCREs might also interact with other promoters to regulate the expression of other genes. Further experiments are needed to determine the precise role of these pCREs.

H3K27ac analysis in chordoma, a cancer of the spinal column that *TBXT* is also implicated in [49, 50], revealed a larger 1.5 Mb active region surrounding *TBXT*, harboring two SEs associated with *TBXT* [17]. While the identified lung cancer pCREs did not overlap the *TBXT* SEs of chordoma, the upstream pCRE fell within the 1.5 Mb active region identified in chordoma. This may suggest that there may be shared elements within this broader active region between *TBXT* in H460 lung cancer cells and chordoma.

Two pCREs with high H3K27ac enrichment were analyzed for transcription factor binding motif enrichment. Motifs for forkhead box, zinc finger, and Maf protein families, all implicated in cancer, were identified. For instance, FOXH1 and FOXQ1 in lung, colorectal, and breast cancer promote EMT by controlling proliferation, migration, and invasion through downregulation of E‐cadherin [27, 28, 29]. Maf proteins [30, 31], in complex with BACH1 and 2 [51] are also involved in activating EMT in breast and lung cancer. TBXT itself also drives EMT and cancer progression in lung cancer [7, 13]. While TBXT can autoregulate its expression [17, 18], we did not identify T‐box binding sites in these pCREs, suggesting autoregulation may not occur through these specific regions. Future studies should investigate whether FOX and MAF proteins regulate *TBXT* expression in H460 cells and how this impacts cell migration and invasion. Further insights into *TBXT* regulation may be gained by evaluating the *TBXT* regulatory map in resected tumors and patient‐matched samples.

As the epigenome is dynamic, biological functional validation will be crucial in elucidating the role of the identified pCREs in *TBXT* regulation, including their potential involvement in MAPK signaling pathways in lung cancer [16].

## 5 Conclusions

While our findings lack functional validation, our data still highlight putative genomic control regions that correlate with the expression of *TBXT*. In vivo testing and validation of these regions in primary lung tumor samples are necessary to fill the gaps. This approach will refine the understanding of the TBXT regulatory network and provide a platform to investigate epigenetic modifiers and chromatin regulators that are linked to lung cancer. Ultimately, this knowledge will contribute to uncovering mechanisms driving lung cancer disease progression.

## Author Contributions

**Reuben M. Yaa:** funding acquisition, investigation, methodology, project administration, formal analysis, data curation, visualization, writing – original draft. **Brian M. Schilder:** formal Analysis, data curation, visualization, software, writing – original draft. **Rafael D. Acemel:** software, resources. **Fiona C. Wardle:** conceptualization, project administration, methodology, resources, supervision, writing – review and editing.

## Ethics Statement

The study did not include patient or animal samples. No approval was required.

## Conflicts of Interest

The authors declare no conflicts of interest.

## Supporting information


**Figure S1.** *TBXT* expression is maintained in H460, while absent in A549 and H358 cells. (A) *TBXT* expression (172 bp), measured by RT‐PCR, is detected in H460 cells, but not in A549 and H358 cells. *GAPDH* expression (121 bp) a housekeeping gene was a control and was detected in all cell lines (*n* = 3). (B) TBXT protein is detected, by western blot, in H460 cells, but not in A549 and H358 cells. H3 protein as a control was detected in all the cell lines (*n* = 3). (C) Immunostaining also detects TBXT protein in the nucleus (stained with DAPI) of H460 cells, but not in A549 and H358 cells (*n* = 3). The scale bar is 50 μM. (D) TBXT nuclear fluorescent intensity is only measurable in H460 with no detectable levels in A549 and H358 cells. DAPI nuclear fluorescent intensities were insignificantly different between the cell lines.

**Figure S2.** 4C seq fragment count between the samples. (A) Merged normalized 4C‐seq profiles. (B) 4C replicates correlations between the replicates. Scale represents normalized counts of the interactions. (C) Heatmap and dendrogram of pairwise correlation matrix between the samples on merging. Scale is Pearson's correlation.

**Figure S3.** Clustering and correlation of ChIP‐seq data. Pairwise correlation heatmap of ChIP‐seq peaks at (A) chr6 and (B) +/− 1 Mb around *TBXT* locus after rebinning each set of genomic ranges using EpiCompare [44]. Peaks clustered as sample type and histone class (H3K27ac, H3K4me3 for active marks, and H3K27me3, H3K9me3 for repressive marks).

**Table S1.** 4C sequencing primers.

**Table S2.** ChIP‐seq metadata including SRA accession numbers.

**Table S3.** Annotation of the pCREs.

**Table S4.** Motif discovery.

## Data Availability

The data that support the findings of this study are openly available in NCBI's Gene Expression Omnibus at https://www.ncbi.nlm.nih.gov/geo/, reference number GSE274316.
